# Immediate Versus Salvage Postoperative Radiotherapy in High-Risk Prostate Cancer Patients: A Critical Review

**DOI:** 10.7759/cureus.27678

**Published:** 2022-08-04

**Authors:** Soufiane Berhili, Mohammed Amine Guerrouaz, Fatima Zahra Terrab, Mohammed Moukhlissi, Loubna Mezouar

**Affiliations:** 1 Radiation Oncology, Faculty of Medicine, Mohammed First University, Oujda, MAR; 2 Radiation Oncology, Hassan II Center of Oncology, Mohammed VI University Hospital, Oujda, MAR; 3 Radiation Therapy, Centre Hospitalier Universitaire Mohammed Vi, Oujda, MAR

**Keywords:** outcomes, early salvage radiotherapy, immediate adjuvant radiotherapy, radical prostatectomy, high-risk prostate cancer

## Abstract

Radical prostatectomy in high-risk prostate cancer patients has long been followed by immediate adjuvant radiotherapy (IART) to increase biochemical relapse-free survival. However, the increased urinary and digestive radio-induced toxicities have raised questions about the safety of delaying radiotherapy until the occurrence of biochemical or clinical relapse. Recently, early salvage radiotherapy (ESRT) has been compared to IART, and results found equivalence in terms of efficiency outcomes, but increased toxicity was noted in patients receiving IART, leading to the proposal of ESRT as the new standard of care in high-risk patients after surgery. However, several confounding points are discussed in the present review regarding the methodology and results of these recent trials. Further follow-up is necessary to detect possible long-term advantages of one radiotherapy timing over the other.

## Introduction and background

Prostate cancer is the most common cancer diagnosis in men worldwide, with a total of approximately 1,280,000 new cases and 359,000 deaths reported in 2018 [1-3]. High-risk prostate cancer patients are treated either with external beam radiotherapy (RT) with long-course androgen deprivation therapy or with radical prostatectomy (optionally associated with pelvic lymphadenectomy) followed by immediate adjuvant radiotherapy (IART) when adverse risk factors are present [4].

Based on existing data, both strategies are equivalent in their rate of tumor control, but each results in different side effects [5]. Surgery often causes urinary incontinence, erectile dysfunction (particularly when no nerve-sparing techniques are used), urethral strictures, and inguinal hernias. In contrast, RT with androgen deprivation therapy often results in increased bowel and bladder irritation and high rates of bone and cardiovascular events [6-8]. Determining a patient’s treatment strategy depends not only on their life expectancy and associated health conditions but also on their preferences after having been sufficiently informed about the possible side effects of each therapy.

In high-risk patients initially treated by surgery, numerous adverse risk factors like high preoperative serum levels of prostate-specific antigen (PSA), pT3 stage, positive surgical margins, and Gleason score ≥ 8 may indicate a high probability of residual tumor cells within the prostate bed. This may predispose a patient to disease recurrence 6 to 8 years after surgery, with an average overall survival (OS) limited to approximately 10 years [9-12].

To consolidate disease control, patients undergoing surgery who have one or more of the abovementioned risk factors should receive IART to eradicate the remaining cancer cells in the prostate bed [13]. Many randomized trials have demonstrated that IART significantly increased the rates of biochemical relapse-free survival (BRFS), local control (LC), and disease-free survival (DFS) [9-11]. Therefore, IART has been widely recommended within six months after surgery in high-risk prostate cancer patients [4,14].

For several years, practitioners raised concerns about the toxicities their patients experienced after undergoing RT and questioned the optimal time to propose adjuvant RT. Delaying RT until the disease relapses (early salvage RT) appeared to be more protective against radiation-induced toxicities, but evidence of its safety in disease control remained lacking. The results of a recent meta-analysis of three randomized trials comparing IART and ESRT demonstrated that both strategies were equivalent in disease control although significantly lower toxicity rates were noted in patients who received ESRT compared to those who received IART [15-17].

In this article, we aimed to review the existing evidence for the timing of postoperative RT in high-risk prostate cancer patients, both immediately after surgery and in the early salvage setting after disease relapse. In addition, we discussed specific methodological procedures applied in recent trials with particular regard to surgical techniques and radiation modalities. With our analysis, we intend to reflect upon the robustness of the conclusions that recent studies have drawn.

## Review

Trials comparing IART and the “wait-and-see” policy

International guidelines currently recommend the use of IART after radical prostatectomy in high-risk prostate cancer patients, primarily to prevent biochemical relapse [4,18]. These recommendations are based on the results of three major randomized trials that have demonstrated the benefit of IART over the “wait-and-see” (WS) policy: the South-West Oncology Group (SWOG) 8794 trial, the European Organisation for Research and Treatment of Cancer (EORTC) 22911 trial, and the German Cancer Society (ARO) 96-02 trial. The results of these trials indicate that IART significantly improved BRFS, LC, and DFS (and even OS in the SWOG trial) in patients with adverse risk factors such as high PSA serum levels, Gleason scores ≥ 8, pT3 stage, and positive surgical margins [9-11].

Indeed, patients in these trials who presented adverse risk factors had a 10-year biochemical relapse rate as high as 75% after radical prostatectomy alone when IART was omitted [19]. Nearly one-third of patients assigned to the WS group required RT for biochemical and/or clinical relapse during follow-up: 33.1 % in the SWOG 8794 trial and 31.4 % in the EORTC 22911 trial [9-10]. However, these trials also found significantly higher rates of severe genitourinary toxicity in patients receiving IART compared to those followed by the WS policy. Table 1 displays the main 10-year efficiency and toxicity results of these trials.

**Table 1:** Main 10-year results of the trials comparing IART with WS policy after surgery in high-risk prostate cancer patients 10y BFRS: 10-year biochemical relapse-free survival; 10y OS: 10-year overall survival; G.I.: gastrointestinal; n: number of patients; IART: immediate adjuvant radiotherapy; WS: "wait-and-see" policy; p: statistical significance set at 0,05; NA: not applicable; SWOG: South-West Oncology Group; EORTC: European Organisation for Research and Treatment of Cancer; ARO: German Cancer Society

Trial *(year) *Patients	10y BRFS			10y OS			Grade 2-4 Urinary Toxicity			Grade 2-4 G.I. Toxicity		
	IART	WS	p	IART	WS	p	IART	WS	p	IART	WS	p
SWOG 8794* (2009)*, n=425 [9]	64%	34.9%	<0.001	59%	48%	0.023	17.8%	9.5%	0.02	3.3%	0%	0.02
EORTC 22911 *(2012),* n=1005 [10]	60.6%	41.1%	<0.0001	76.9%	80.7%	0.2	21.3%	13.5%	0.003	2.5%	1.9%	0.47
ARO 96-02*(2014)*, n=307 [11]	56%	35%	<0.0001	84.5%	87.4%	0.59	2.7%	0%	NA	1.3%	0%	NA

Nevertheless, and despite the increased toxicity, most practitioners offered IART to their patients for fear of early disease relapse. Therefore, IART has been the standard of care for high-risk prostate cancer patients after surgery for more than a decade [4,18].

Trials and meta-analysis comparing IART and ESRT

In the SWOG 8794, EORTC 22911, and ARO 96-02 trials, radical prostatectomy followed by IART was compared to radical prostatectomy alone followed by the WS policy. These trials did not report follow-up of patients after receiving salvage RT for disease relapse in the WS arm. Given the high toxicity rate caused by IART, some clinicians have started to propose close monitoring for frail patients and the delay of RT until disease relapse. According to available data as of September 2020, it remained unclear whether ESRT after biochemical or clinical relapse could provide disease control rates equal to IART after radical prostatectomy for high-risk patients. All previous comparisons between the two timings of RT were conducted retrospectively.

Trials comparing IART and ESRT

Recently, three large phase-III randomized controlled trials comparing IART and ESRT after surgery in high-risk prostate cancer patients have been reported: the RADICALS-RT, GETUG-AFU 17, and RAVES trials, which were simultaneously published with their planned meta-analyses of aggregate data [15-17,20].

Five years after randomization in the RADICALS-RT and GETUG-AFU17 trials, 223 patients out of 699 (32%) and 114 patients out of 212 (54%) assigned to the ESRT group did receive salvage RT for disease relapse, respectively [15-16]. It should be noted that the RADICALS-RT trial was by far the largest of the three trials included in the meta-analysis with roughly two-thirds of the total number of patients, followed by the GETUG-AFU17 trial with almost 20% of patients.

In these recent trials, the primary efficiency outcome (BRFS) was similar between the IART and ESRT groups. However, significant differences in late genitourinary and gastrointestinal toxicity rates were reported in favor of the ESRT group. Table 2 displays the main five-year efficiency and toxicity results of the trials comparing IART versus ESRT [15-17].

**Table 2:** Main 5-year results of trials comparing IART and ESRT after surgery for high-risk prostate cancer patients G. I.: gastrointestinal; NR: not reported; BRFS: biochemical relapse-free survival; IART: immediate adjuvant radiotherapy; ESRT: early salvage radiotherapy; * as the published 5yr-event-free survival rate comprised all types of events; the BRFS was calculated from the trial results (7 + 6 biochemical relapse / 212 in the IART group, and 13 + 9 / 212 in the ESRT group); ** grades 1 and 2 toxicity were combined in the RADICAL-RT results

Trial *(year),* Patients	5yr BRFS			5yr OS			Grade 2–4 Urinary Toxicity			Grade 2–4 G. I. Toxicity		
	IART	ESRT	p	IART	ESRT	p	IART	ESRT	p	IART	ESRT	p
RADICALS-RT *(2020), *n=1396 [15]	,85%	88%	0.56	NR	96%	NR	**	**	NR	**	**	NR
GETUG-AFU17 *(2020)*, n=424 [16]	93.9%*	89.7%*	NR	96%	99%	0.25	27%	7%	<0.001	8%	5%	0.24
RAVES *(2020)*, n=333 [17]	86%	89%	0.086	99%	98%	NR	70%	54%	0.002	14%	10%	0.53

Meta-analysis results

The ARTISTIC meta-analysis included 2,153 patients from the three above-mentioned trials who were randomly and equally divided into an IART group and an ESRT group. This meta-analysis was designed to give more than 99% power to detect a 10% difference in event-free survival (EFS) between IART and ESRT [20]. Most patients had either a pT3a-b stage (79.8 %), extra-capsular extension (76.9%), or positive surgical margins (70.9%).

Out of the 1,075 patients in the ESRT group, 421 (39.1%) received salvage RT (ESRT) for biochemical relapse [20]. It should be noted that the “biochemical relapse” was differently defined among the three trials: it corresponded to a PSA level > 0.1 ng/ml or three consecutive rises (even if below 0.1 ng/ml) in the RADICALS-RT trial, a PSA level > 0.2 ng/ml and rising in the GETUG-AFU17 trial, and a PSA level > 0.2 ng/ml in the RAVES trial [15-17].

The meta-analysis indicated a 5-year EFS rate of approximately 88% in both the IART and the ESRT groups, with only a 1% nonsignificant absolute difference (95% CI −2 to 3) between them [20]. Delaying RT until biochemical relapse resulted in avoiding adjuvant RT for almost 60% of patients in the ESRT group, with a significant decrease in genitourinary and gastrointestinal toxicities [20]. In addition, this small difference in EFS (BRFS) hardly entails a survival benefit, based on the previously reported trials that compared IART and the WS policy wherein OS did not improve despite BRFS improvement in both the EORTC 22911 and the ARO 96-02 trials (Table 1). Even in subgroup analyses, the meta-analysis failed to find evidence to suggest that IART had a variable effect on EFS according to preoperative PSA, Gleason score, surgical margins, or seminal vesicle involvement [20].

From a global perspective, this meta-analysis provided consistent evidence of the effects of RT timing after radical prostatectomy in high-risk prostate cancer patients. The authors concluded that the systematic administration of IART following radical prostatectomy (when adverse risk factors are present) does not provide improvement in PSA-driven EFS. Nevertheless, when each trial is scrutinized separately, several concerns arise and the robustness of these findings must be reconsidered.

Closer look at trials comparing IART and ESRT

We conducted a thorough review of the “Patients and Methods” and “Results” sections of the three papers that compared IART and ESRT, which formed the source of the two groups of patients included in the meta-analysis. This allowed us to note several biasing factors regarding not only the surgical and radiation methodologies adopted but also the results of late toxicities and metastasis-free survival.

Extent of surgery

Six hundred and nineteen patients out of 1,396 (44%) in the RADICALS-RT trial and 119 out of 424 (28%) in the GETUG-AFU17 trial - representing 34% of all patients included in the meta-analysis - have not undergone pelvic lymph node dissection during surgery [15-16]. Although these patients were distributed equally and randomly between the IART and ESRT groups, the number of patients assigned to the ESRT group who had not undergone pelvic dissection may have contributed to the low toxicity rate in this group, considering that the patients who remained relapse-free underwent prostatectomy alone without needing salvage RT (297 of 699 (42%) and 61 of 212 (29%) patients in the RADICALS-RT and GETUG-AFU17 trials, respectively).

Forgoing pelvic lymph node dissection during surgery may avoid pelvic toxicities (urinary or digestive) in the ESRT group more so than in the IART group. This can be explained by the fact that pelvic radiation in an immediate postoperative setting (IART) markedly reduces normal pelvic tissue healing after pelvic dissection than after a prostatectomy alone, whereas in the ESRT group, pelvic radiation was performed several months or years after surgery (i.e., after pelvic tissue has normally healed), even when pelvic dissection has been performed.

Radiotherapy fractionation

It is also important to highlight the permission to use hypo-fractionated RT (52.5 Gy in 20 fractions) in the RADICALS-RT trial for both the IART and ESRT groups [15]. As previously noted, patients in the RADICALS-RT trial represented two-thirds of all patients included in the meta-analysis. Although supported by high-level evidence in a definitive setting for prostate cancer patients, moderately hypo-fractionated schemes find less agreement in a postoperative setting [21-22]. Indeed, existing RT guidelines recommend doses per fraction of 1.8 Gy instead of 2 Gy for postoperative indications because it is radiobiologically recognized that the lower the dose is per fraction, the greater the protection is against late radiation toxicity [4].

Thus, radio-induced fibrosis is believed to be more extensive with hypo-fractionated IART when postoperative healing is not completely achieved, and even more important if a pelvic lymph node dissection is performed as discussed above. This may have contributed to the high toxicity rates observed in patients who received IART compared to those in the ESRT group in the RADICALS-RT trial.

Long-term toxicities

Diagrams displaying the results of the RADICALS-RT trial illustrate the evolution over time of urinary and digestive toxicities in patients receiving IART and ESRT, with statistical significance calculated for each year of follow-up. Upon analyzing the data, we noted that the significant difference between IART and ESRT regarding urinary incontinence observed one year after randomization declined and became nonsignificant after four years of follow-up (p = 0.0023 at 1 year vs. p = 0.073 at 5 years). A similar evolution was noted for fecal incontinence between the two study groups. Although it was significantly increased (p < 0.0001) at one year, fecal incontinence in the IART group gradually became nonsignificantly different (p = 0.084) from the ESRT group at five years post-randomization [15].

Therefore, we concluded that although bladder and bowel functions deteriorated more in the IART group in early assessments, the toxicity curves of the two groups converged over the years. The differences in urinary and fecal incontinence between the IART and ESRT groups became nonsignificant after long-term follow-up.

Distant metastasis-free survival

Distant metastasis-free survival (DMFS) was not directly mentioned in the results of the trials. It is a major indicator of survival similar to other reported parameters, such as BRFS or LC, especially in high-risk diseases known to have a relatively high potential for dissemination. This interesting outcome indicator could tip the balance in favor of one group over the other.

The GETUG-AFU17 trial was the second largest trial included in the meta-analysis, containing almost 20% of the total number of patients. A thorough reading of its results allowed us to extract data that could be used to calculate DMFS at five years of follow-up. Three out of 212 patients (1.4%) in the IART group developed distant metastasis versus eight out of 212 (3.8%) in the ESRT group (almost three times greater). Thus, the calculated five-year DMFS for the IART and ESRT groups was 98.6% and 96.2%, respectively [16].

This result indicates a possible trend toward DMFS benefits in favor of the IART group. However, according to the authors of the GETUG-AFU17 trial, the data used were not yet mature enough to confirm the statistical significance of this survival parameter. Longer follow-up can provide evidence of a survival benefit in favor of IART or ESRT.

## Conclusions

Given the satisfactory results achieved by ESRT, the current National Comprehensive Cancer Network prostate cancer guidelines must be revised, particularly in the “Post-Operative Observation” chapter, wherein “curative ESRT” should replace the term “palliative therapy” in the proposed options of postoperative treatment strategies. Nevertheless, it may not be wise to rush into rapid practice-changing conclusions regarding the best timing to deliver postoperative RT in high-risk prostate cancer patients, as further follow-up is needed to detect the possible long-term advantages of one RT timing over the other.

## References

[1] Jemal A, Center MM, DeSantis C, Ward EM (2010). Global patterns of cancer incidence and mortality rates and trends. Cancer Epidemiol Biomarkers Prev.

[2] Torre LA, Bray F, Siegel RL, Ferlay J, Lortet-Tieulent J, Jemal A (2015). Global cancer statistics, 2012. CA Cancer J Clin.

[3] Mattiuzzi C, Lippi G (2019). Current cancer epidemiology. J Epidemiol Glob Health.

[4] (2022). NCCN Clinical Practice Guidelines in OncologyTM. Prostate cancer V.3.2022. https://www.nccn.org/professionals/physician_gls/pdf/prostate.pdf.

[5] Barocas DA, Alvarez J, Resnick MJ (2017). Association between radiation therapy, surgery, or observation for localized prostate cancer and patient-reported outcomes after 3 years. JAMA.

[6] Moncada I, López I, Ascencios J, Krishnappa P, Subirá D (2019). Complications of robot assisted radical prostatectomy. Arch Esp Urol.

[7] Bratu O, Oprea I, Marcu D, Spinu D, Niculae A, Geavlete B, Mischianu D (2017). Erectile dysfunction post-radical prostatectomy - a challenge for both patient and physician. J Med Life.

[8] Mallick S, Madan R, Julka PK, Rath GK (2015). Radiation induced cystitis and proctitis - prediction, assessment and management. Asian Pac J Cancer Prev.

[9] Thompson IM, Tangen CM, Paradelo J (2009). Adjuvant radiotherapy for pathological T3N0M0 prostate cancer significantly reduces risk of metastases and improves survival: long-term followup of a randomized clinical trial. J Urol.

[10] Bolla M, Poppel HV, Tombal B (2012). Postoperative radiotherapy after radical prostatectomy for high-risk prostate cancer: long-term results of a randomised controlled trial (EORTC trial 22911). Lancet.

[11] Wiegel T, Bartkowiak D, Bottke D (2014). Adjuvant radiotherapy versus wait-and-see after radical prostatectomy: 10-year follow-up of the ARO 96-02/AUO AP 09/95 trial. Eur Urol.

[12] Dotan ZA, Bianco FJ Jr, Rabbani F (2005). Pattern of prostate-specific antigen (PSA) failure dictates the probability of a positive bone scan in patients with an increasing PSA after radical prostatectomy. J Clin Oncol.

[13] Zietman AL, Bae K, Slater JD (2010). Randomized trial comparing conventional-dose with high-dose conformal radiation therapy in early-stage adenocarcinoma of the prostate: long-term results from Proton Radiation Oncology Group/American College of Radiology 95-09. J Clin Oncol.

[14] Golbari NM, Katz AE (2017). Salvage therapy options for local prostate cancer recurrence after primary radiotherapy: a literature review. Curr Urol Rep.

[15] Parker CC, Clarke NW, Cook AD (2020). Timing of radiotherapy after radical prostatectomy (RADICALS-RT): a randomised, controlled phase 3 trial. Lancet.

[16] Sargos P, Chabaud S, Latorzeff I, & al (2020). Adjuvant radiotherapy versus early salvage radiotherapy plus short-term androgen deprivation therapy in men with localised prostate cancer after radical prostatectomy (GETUG-AFU 17): a randomised, phase 3 trial. Lancet Oncol.

[17] Kneebone A, Fraser-Browne C, Duchesne GM (2020). Adjuvant radiotherapy versus early salvage radiotherapy following radical prostatectomy (TROG 08.03/ANZUP RAVES): a randomised, controlled, phase 3, non-inferiority trial. Lancet Oncol.

[18] Parker C, Castro E, Fizazi K (2020). Prostate cancer: ESMO Clinical Practice Guidelines for diagnosis, treatment and follow-up. Ann Oncol.

[19] Nguyen CT, Reuther AM, Stephenson AJ, Klein EA, Jones JS (2009). The specific definition of high risk prostate cancer has minimal impact on biochemical relapse-free survival. J Urol.

[20] Vale CL, Fisher D, Kneebone A (2020). Adjuvant or early salvage radiotherapy for the treatment of localised and locally advanced prostate cancer: a prospectively planned systematic review and meta-analysis of aggregate data. Lancet.

[21] Chin S, Fatimilehin A, Walshaw R (2020). Ten-year outcomes of moderately hypofractionated salvage postprostatectomy radiation therapy and external validation of a contemporary multivariable nomogram for biochemical failure. Int J Radiat Oncol Biol Phys.

[22] Viani GA, Gouveia AG, Leite ET, Moraes FY (2022). Moderate hypofractionation for salvage radiotherapy (HYPO-SRT) in patients with biochemical recurrence after prostatectomy: a cohort study with meta-analysis. Radiother Oncol.

